# Partition of Neutral Molecules and Ions from Water to *o*-Nitrophenyl Octyl Ether and of Neutral Molecules from the Gas Phase to *o*-Nitrophenyl Octyl Ether

**DOI:** 10.1007/s10953-018-0717-0

**Published:** 2018-02-16

**Authors:** Michael H. Abraham, William E. Acree, Xiangli Liu

**Affiliations:** 1 Department of Chemistry, University College London, 20 Gordon St, London, WC1H 0AJ UK; 2 Department of Chemistry, University of North Texas, 1155 Union Circle Drive #305070, Denton, TX 76203-5017 USA; 3 School of Pharmacy and Medical Sciences, Faculty of Life Sciences, University of Bradford, Bradford, BD7 1DP UK

**Keywords:** *o*-Nitrophenyl ocytl ether, Partition coefficients, Absolv descriptors, Ionic species, Linear free energy relationships

## Abstract

We have set out an equation for partition of 87 neutral molecules from water to *o*-nitrophenyl octyl ether, NPOE, an equation for partition of the 87 neutral molecules and 21 ionic species from water to NPOE, and an equation for partition of 87 neutral molecules from the gas phase to NPOE. Comparison with equations for partition into other solvents shows that, as regards partition of neutral (nonelectrolyte) compounds, NPOE would be a good model for 1,2-dichloroethane and for nitrobenzene. In terms of partition of ions and ionic species, NPOE is quite similar to 1,2-dichloroethane and not far away from other aprotic solvents such as nitrobenzene.

## Introduction

The partition of compounds from water to organic phases is of extreme importance in extraction studies and in the purification of organic compounds. In particular, the interactions between compounds and various possible solvents for extraction is of crucial importance. We have set out equations that encode such interactions for the partition of organic compounds from water to some 50 different solvents [1–6]. Inspection of these equations then affords a simple and practical method for the choice of a solvent in order to selectively extract a given compound from a mixture. More recently, we have extended this work to include the extraction of permanent ions (K^+^ or Cl^−^ for example) and of ionic species (specifically anions from the deprotonation of carboxylic acids and cations from the protonation of nitrogen bases) [7]. The number of solvents for which we have an equation for neutral molecules and ions is still quite small, although the method has been used to obtain equations for partition from water to water–ethanol mixtures [8] and to water–methanol mixtures [9] and extended into equations for permeation from saline solutions into brains [10], microsomal binding [11], artificial membrane retention factors [12], human intestinal absorption [13], partition into cerasome [14] and human skin permeability [15]. Quite recently, Davis and Di Toro [16] have examined partition of ionic species into organic solvents using quantum chemically calculated parameters.

Some time ago we examined partition of neutral molecules and ionic species from water into *o*-nitrophenyl octyl ether, NPOE [17], but could use only 55 out of the partition coefficients for 88 neutral compounds that were reported [18], and could use only partition coefficients for 15 ions and ionic species. Since then, we have determined descriptors for all the 88 neutral compounds, and have collected partition coefficients for 23 ions [19–21]. In view of the comparatively small number of solvents for which we have equations for neutral molecules and ions, we thought it useful to construct an up-to-date equation for the water–NPOE system, and then to be able to compare this system more rigorously with the other systems that we have studied. Such comparisons have in the past identified possible safe solvent alternatives to replace several of the more hazardous organic solvents used in industrial manufacturing processes, and have found organic partitioning systems that could possibly mimic some of the biological responses. An equation describing transfer of both neutral molecules and ions from water to NPOE could also be used to estimate the dissociation constants of substituted benzoic acids and substituted phenols, as well as the dissociation constants of substituted anilinium and substituted pyridinium cations, in NPOE.

## Methodology

For partition of neutral molecules from water to another solvent we use our well-known linear free energy relationships, LFERs, Eqs. 1 and 2 [22, 23]:

1 $$ { \log }_{ 10}\;P = c + e{\user2{E}} + {\mathbf{s}}{\user2{S}} + a{\user2{A}}+ b{\user2{B}}+ v{\user2{V}} $$

2 $$ {\log}_{10}\;K = c + e{\user2{E}} + {\mathbf{s}}{\user2{S}} + a{\user2{A}}+ b{\user2{B}}+ l{\user2{L}} $$

In Eq. 1, the dependent variable is log_10_ *P*, where *P* is the water-to-solvent partition coefficient for a series of nonelectrolytes in a given water to solvent system. In Eq. 2, the dependent variable is log_10_ *K*, where *K* is the gas phase to solvent system partition coefficient. The independent variables are descriptors as described previously [20–23]. ***E*** is the nonelectrolyte (or solute) excess molar refractivity in units of (cm^3^·mol^−1^)/10, ***S*** is the solute dipolarity/polarizability, ***A*** and ***B*** are the overall or summation solute hydrogen bond acidity and basicity, ***V*** is the solute McGowan characteristic volume in units of (cm^3^·mol^−1^)/100, and ***L*** is log_10_ *K*_16_, where *K*_16_ is the gas to hexadecane partition coefficient at 298 K. The coefficients in Eq. 1 are given in Table 1.

**Table 1:** Coefficients in Eqs. 1 and 4

Solvent, Eq. 1	No	***c***	***e***	***s***	***a***	***b***	***v***	***j*** **+**	***j*** ^**−**^
Methanol	1	0.276	0.334	− 0.714	0.243	− 3.320	3.549	− 2.609	3.027
Ethanol	2	0.222	0.471	− 1.035	0.326	− 3.596	3.857	− 3.170	3.085
Propan-1-ol	3	0.139	0.405	− 1.029	0.247	− 3.767	3.986	− 3.077	2.834
Butan-1-ol	4	0.165	0.401	− 1.011	0.056	− 3.958	4.044	− 3.605	2.685
Wet octan-1-ol	5	0.088	0.562	− 1.054	0.034	− 3.460	3.814	− 3.023	2.580
Hexan-1-ol	6	0.115	0.492	− 1.164	0.054	− 3.971	4.131	− 3.100	2.940
Propan-2-ol	7	0.099	0.344	− 1.049	0.406	− 3.827	4.033	− 3.896	2.889
t-Butanol	8	0.211	0.171	− 0.947	0.331	− 4.085	4.109	− 4.455	2.953
Ethylene glycol	9	− 0.270	0.578	− 0.511	0.715	− 2.619	2.729	− 1.300	2.363
1,2-Propylene glycol	10	− 0.149	0.754	− 0.966	0.684	− 3.134	3.247	− 1.381	3.057
Formamide	11	− 0.171	0.070	0.308	0.589	− 3.152	2.432	− 3.152	2.432
Dimethylformamide	12	− 0.305	− 0.058	0.343	0.358	− 4.865	4.486	− 3.605	0.415
Dimethylacetamide	13	− 0.271	0.084	0.209	0.915	− 5.003	4.557		0.286
Acetonitrile	14	0.413	0.077	0.326	− 1.566	− 4.391	3.364	− 2.234	0.101
Nitromethane	15	0.023	− 0.091	0.793	− 1.463	− 4.364	3.460		− 0.149
*N*-Methylpyrrolidinone	16	0.147	0.532	0.275	0.840	− 4.794	3.674	− 1.797	0.105
Dimethylsulfoxide	17	− 0.194	0.327	0.791	1.260	− 4.540	3.361	− 3.387	0.132
Propanone	18	0.313	0.312	− 0.121	− 0.608	− 4.753	3.942	− 2.288	0.078
1,2-Dichloroethane	19	0.183	0.294	− 0.134	− 2.801	− 4.291	4.180	− 3.429	− 0.025
Propylene carbonate	20	0.004	0.168	0.504	− 1.283	− 4.407	3.424	− 1.989	0.341
Sulfolane	21	0.000	0.147	0.601	− 0.318	− 4.541	3.290	− 1.200	− 0.792
Nitrobenzene	22	− 0.152	0.525	0.081	− 2.332	− 4.494	4.187	− 3.373	0.777
Benzonitrile	23	0.097	0.285	0.059	− 1.605	− 4.562	4.028	− 2.729	0.136
Chlorobenzene	24	0.065	0.381	− 0.521	− 3.183	− 4.700	4.614	− 4.536	− 1.486
Tetrahydrofuran	25	0.223	0.363	− 0.384	− 0.238	− 4.932	4.450	− 2.278	− 2.132
NPOE (this work)	26	0.182	0.631	− 0.447	− 2.254	− 3.973	3.559	− 2.342	0.444

Solvent, Eq. 2	No	***c***	***e***	***s***	***a***	***b***	***l***
Methanol	1	− 0.039	− 0.338	1.317	3.826	1.396	0.773
Ethanol	2	0.017	− 0.232	0.867	3.894	1.192	0.846
Propan-1-ol	3	− 0.042	− 0.246	0.749	3.888	1.076	0.874
Butan-1-ol	4	− 0.004	− 0.285	0.768	3.705	0.879	0.890
Wet octan-1-ol	5	− 0.222	0.088	0.701	3.473	1.477	0.851
Hexan-1-ol	6	− 0.014	− 0.205	0.583	3.621	0.891	0.913
Propan-2-ol	7	− 0.048	− 0.324	0.713	4.036	1.055	0.884
t-Butanol	8	0.053	− 0.443	0.699	4.026	0.882	0.907
Ethylene glycol	9	− 0.887	0.132	1.657	4.457	2.355	0.565
1,2-Propylene glycol	10	− 0.607	0.239	1.008	4.278	1.755	0.706
Formamide	11	− 0.800	0.310	2.292	4.130	1.933	0.442
Dimethylformamide	12	− 0.391	− 0.869	2.107	3.774	0.000	1.011
Dmethylacetamide	13	− 0.308	− 0.736	1.802	4.361	0.000	1.028
Acetonitrile	14	− 0.007	− 0.595	2.461	2.085	0.418	0.738
Nitromethane	15	− 0.340	− 0.297	2.689	2.193	0.514	0.728
*N*-Methylpyrrolidinone	16	− 0.128	− 0.029	2.217	4.429	0.000	0.777
Dimethylsulfoxide	17	− 0.556	− 0.223	2.903	5.037	0.000	0.719
Propanone	18	0.127	− 0.387	1.733	3.060	0.000	0.866
1,2-Dichloroethane	19	0.017	− 0.337	1.600	0.774	0.637	0.921
Propylene carbonate	20	− 0.356	− 0.413	2.587	2.207	0.455	0.719
Sulfolane	21	− 0.414	0.084	2.396	3.144	0.420	0.684
Nitrobenzene	22	− 0.296	0.092	1.707	1.147	0.443	0.912
Benzonitrile	23	− 0.075	− 0.341	1.798	2.030	0.291	0.880
Chlorobenzene	24	0.064	− 0.399	1.151	0.313	0.171	1.032
Tetrahydrofuran	25	0.189	− 0.347	1.238	3.289	0.000	0.982
NPOE (this work)	26	− 0.104	0.290	1.333	1.306	0.967	0.759

The experimental determination of the descriptors for neutral compounds to use in Eqs. 1 and 2 has been reviewed several times [20–25]. These experimental descriptors are available both commercially [26] and in the public domain [27], and descriptors can also be calculated for nonelectrolytes [26, 27]. It is very useful if we can calculate some of the descriptors. The ***E***-descriptor can be obtained from a refractive index at 293 K (for liquid solutes), or can be calculated from an estimated refractive index [26]. Both available software programs [26, 27] give calculated values of ***E***. The ***V***-descriptor can easily be calculated from its molecular formula [22, 28] and is calculated by the two software programs [26, 27]. A valuable ‘extra’ descriptor is log_10_ *K*_w_ where *K*_w_ is the gas-to-water partition coefficient at 298 K; note that *K*_w_ is dimensionless. Descriptors for the 88 non-electrolytes are in Table 2, together with values of the water–NPOE partition coefficient [18], as log_10_ *P*_npoe_, values of log_10_ *K*_w_ and corresponding values of log_10_ *K*_npoe_ obtained through Eq. 3.

**Table 2:** Descriptors for non-electrolytes and corresponding values of log_10_ *K*_w_, log_10_ *P*_npoe_ and log_10_ *K*_npoe_

Solute	***E***	***S***	***A***	***B***	***V***	***L***	log_10_*K*_w_	log_10_ *P*	log_10_ *K*
								NPOE	NPOE
4-Methylbenzylamine	0.829	0.79	0.15	0.68	1.0980	4.815	4.45	1.80	6.25
4-Methyl-*N*-methylbenzylamine	0.800	0.74	0.14	0.73	1.2389	5.166	4.36	1.48	5.84
4-Methyl-*N*-ethylbenzylamine	0.790	0.75	0.14	0.76	1.3798	5.660	4.41	1.86	6.27
4-Methyl-*N*-propylbenzylamine	0.780	0.70	0.14	0.76	1.5207	6.086	4.20	2.36	6.56
4-Methyl-*N*-butylbenzylamine	0.770	0.68	0.14	0.76	1.6616	6.455	4.01	2.90	6.91
4-Methyl-*N*-pentylbenzylamine	0.760	0.68	0.14	0.75	1.8025	7.081	3.82	3.48	7.30
4-Methyl-*N*-hexylbenzylamine	0.750	0.69	0.13	0.74	1.9434	7.608	3.64	4.07	7.71
4-Methyl-*N*-heptylbenzylamine	0.740	0.69	0.14	0.76	2.0843	8.000	3.67	4.29	7.96
4-Bromobenzoic acid^a^	1.000	1.02	0.63	0.27	1.1067	5.472	4.94	0.82	5.76
3-Chlorobenzoic acid	0.840	0.95	0.63	0.32	1.0541	5.197	5.15	0.94	6.09
4-Chlorobenzoic acid	0.840	1.02	0.63	0.27	1.0541	4.947	4.80	0.88	5.68
4-Iodobenzoic acid	1.310	1.27	0.63	0.30	1.1899	6.487	5.84	1.46	7.30
Carprofen	2.290	1.90	0.95	0.80	1.9349	10.923	11.03	3.01	14.04
Flurbiprofen	1.440	1.45	0.62	0.76	1.8389	8.975	7.95	2.94	10.89
a-Methyl-4-isobutylphenylacetic acid	0.730	0.98	0.59	0.66	1.7771	7.568	5.86	2.72	8.58
Naproxen	1.510	2.02	0.60	0.67	1.7821	9.207	8.80	2.51	11.31
Pirprofen	1.480	1.47	0.60	0.81	1.8477	8.937	8.27	2.61	10.88
Suprofen	1.510	1.89	0.60	0.99	1.9026	9.673	10.17	1.76	11.93
Ketoprofen	1.650	2.26	0.55	0.89	1.9779	10.527	10.46	1.81	12.27
Indomethacin	2.236	1.47	0.58	1.43	2.5299	12.270	11.07	3.03	14.10
Benzoic acid	0.730	0.90	0.59	0.40	0.9317	4.657	5.10	0.50	5.60
Phenylacetic acid	0.730	1.08	0.66	0.57	1.0726	4.960	6.48	0.12	6.60
3-Phenylpropanoic acid	0.750	1.18	0.60	0.60	1.2135	5.616	6.60	0.51	7.11
4-Phenylbutanoic acid	0.760	1.29	0.61	0.57	1.3544	6.204	6.65	1.28	7.93
5-Phenylpentanoic acid	0.770	1.24	0.57	0.60	1.4953	6.589	6.36	1.77	8.13
7-Phenylheptanoic acid	0.790	1.27	0.57	0.62	1.7771	7.599	6.39	2.52	8.91
8-Phenyloctanoic acid	0.790	1.30	0.59	0.65	1.9180	8.132	6.58	2.97	9.55
Antipyrine	1.300	1.83	0.00	1.37	1.4846	7.764	9.74	− 0.03	9.71
Diazepam	2.170	1.78	0.00	1.27	2.0739	11.010	9.22	2.96	12.18
Homatropine	1.400	1.33	0.05	1.76	2.1411	9.689	10.11	1.10	11.21
Nicotine	0.865	0.88	0.00	1.09	1.3710	5.888	5.85	0.60	6.45
5-Ethyl-5-phenylbarbital	1.630	1.72	0.71	1.18	1.6999	8.956	11.32	0.02	11.34
5,5-Diphenylhydantoin	1.713	2.23	0.86	1.00	1.8693	10.236	12.22	0.80	13.02
3-Nitrobenzyl alcohol	1.064	1.44	0.40	0.59	1.0902	5.653	6.74	1.10	7.84
Sulfanilamide	1.500	1.82	0.41	1.19	1.1969	7.010	10.81	− 1.02	9.79
Sulfacetamide	1.480	2.73	0.42	1.30	1.4944	8.730	13.45	− 0.64	12.81
Sulfabenzamide	2.130	2.90	0.45	1.16	1.9613	11.639	13.28	1.05	14.33
Sulfapyridine	2.040	2.23	0.59	1.48	1.7636	10.270	13.74	− 0.21	13.53
Sulfamethazine	2.130	2.53	0.59	1.53	2.0043	11.504	14.63	0.12	14.75
Sulfisomidine	2.130	2.70	0.48	1.76	2.0043	11.470	16.02	− 1.22	14.80
Sulfamethoxypyridazine	2.120	2.95	0.48	1.56	1.9221	11.408	15.48	− 0.08	15.40
Sulfacytine	2.290	2.67	0.32	1.75	2.0630	11.280	15.14	− 0.45	14.69
Sulfadoxine	2.140	2.53	0.40	1.64	2.1217	11.740	14.35	0.69	15.04
Sulfadimethoxine	2.140	2.39	0.51	1.53	2.1217	11.780	13.86	1.34	15.20
Sulfathiazole	2.110	2.57	0.33	1.40	1.6883	10.010	13.38	− 0.38	13.00
Sulfamethoxazole	1.890	2.45	0.62	1.20	1.7244	10.040	13.03	0.52	13.55
Sulfamoxole	1.900	2.13	0.26	1.66	1.8653	9.840	13.00	0.12	13.12
Sulfamethizole	2.140	2.40	0.61	1.44	1.7881	10.530	14.13	− 0.48	13.65
Sulfaphenazole	2.700	2.62	0.52	1.70	2.2324	13.170	15.55	0.98	16.53
Methyl acetate	0.142	0.64	0.00	0.45	0.6057	1.911	2.30	0.28	2.58
Ethyl acetate	0.106	0.62	0.00	0.45	0.7466	2.314	2.16	0.91	3.07
Butyl acetate	0.071	0.60	0.00	0.45	1.0284	3.353	1.94	1.86	3.80
Acetonitrile	0.237	0.90	0.07	0.32	0.4042	1.739	2.85	− 0.22	2.63
Proprionitrile	0.162	0.90	0.02	0.36	0.5451	2.082	2.82	0.27	3.09
*N*,*N*-Dimethylacetamide	0.363	1.38	0.00	0.80	0.7877	3.639	6.04	− 0.64	5.40
Ethanol	0.246	0.42	0.37	0.48	0.4491	1.485	3.67	− 1.29	2.38
Propan-1-ol	0.236	0.42	0.37	0.48	0.5900	2.031	3.56	− 0.76	2.80
Pentan-1-ol	0.219	0.42	0.37	0.48	0.8718	3.106	3.35	0.39	3.74
Hexan-1-ol	0.210	0.42	0.37	0.48	1.0127	3.610	3.23	0.94	4.17
Pentanoic acid	0.205	0.63	0.62	0.45	0.8875	3.227	4.45	− 0.06	4.39
1-Nitrobutane	0.227	0.95	0.00	0.29	0.8464	3.415	2.27	1.99	4.26
Acetophenone	0.818	1.01	0.00	0.48	1.0139	4.501	3.36	2.00	5.36
Nitrobenzene	0.871	1.11	0.00	0.28	0.8906	4.557	3.02	2.44	5.46
1-Chloro-2-nitrobenzene	1.020	1.24	0.00	0.24	1.0130	5.121	3.10	2.88	5.98
Phenylacetonitrile	0.751	1.03	0.00	0.50	1.0120	4.649	3.70	2.12	5.82
Benzylmethylketone	0.748	0.90	0.00	0.70	1.1548	4.726	4.12	1.86	5.98
2-Phenylethyl acetate	0.788	1.10	0.00	0.60	1.3544	5.833	4.03	2.54	6.57
Pyridine	0.631	0.84	0.00	0.52	0.6753	3.022	3.44	0.26	3.70
Acridine	2.356	1.16	0.00	0.60	1.4133	7.814	4.95	3.61	8.56
1-Naphthoic acid	1.460	1.20	0.65	0.49	1.3007	6.910	6.63	1.81	8.44
1-Naphthylamine	1.670	1.27	0.19	0.51	1.1852	6.490	5.34	2.49	7.83
Aniline	0.955	0.96	0.26	0.41	0.8162	3.934	4.30	1.08	5.38
*N*-Ethylaniline	0.945	0.91	0.15	0.43	1.0980	4.865	3.58	2.36	5.94
2-Chloroaniline	1.033	0.92	0.25	0.31	0.9386	4.674	3.60	2.12	5.72
2-Aminobiphenyl	1.600	1.45	0.26	0.44	1.4240	7.516	5.33	3.20	8.53
4,4’-Diaminobiphenyl	1.880	1.96	0.20	1.00	1.5238	8.710	9.35	1.82	11.17
4-Nitroaniline	1.220	1.92	0.46	0.35	0.9904	6.042	7.18	1.52	8.70
Phenol	0.805	0.89	0.60	0.30	0.7751	3.766	4.85	0.58	5.43
3-Chlorophenol	0.909	1.06	0.69	0.15	0.8975	4.773	4.85	1.48	6.33
3-Methylbenzoic acid	0.730	0.89	0.60	0.40	1.0726	4.819	4.98	1.01	5.99
3-Chlorophenylacetic acid	0.840	1.02	0.61	0.57	1.1950	5.561	6.15	0.93	7.08
Benzyl alcohol	0.803	0.87	0.39	0.56	0.9160	4.221	5.10	0.36	5.46
4-Methylbenzyl alcohol	0.810	0.88	0.39	0.60	1.0569	4.584	5.22	1.27	6.49
4-Nitrophenol	1.070	1.72	0.82	0.26	0.9493	5.876	7.81	0.94	8.75
Dimethylsulfoxide	0.522	1.72	0.00	0.97	0.6126	3.459	7.41	− 1.42	5.99
Triethylamine	0.101	0.15	0.00	0.79	1.0538	3.040	2.36	0.81	3.17
*N*,*N*-Dimethylbenzylamine	0.668	0.78	0.00	0.70	1.2389	5.046	3.70	1.64	5.34
Decanoic acid	0.124	0.64	0.62	0.45	1.5920	5.698	3.87	2.94	6.81

^a^Not used in the regressions

3 $$ { \log }_{ 10}\; P_{npoe} = \log_{10}\; K_{npoe} \log_{10}\;K_{w} $$

The determination of equation coefficients in Eq. 1 is a prerequisite for obtaining the corresponding equation for ion transfer, because we have deliberately used Eq. 1 as part of our equation for ion transfer, Eq. 4. In this equation, the coefficients ***c***, ***e***, ***s***, ***a***, ***b*** and ***v*** are set equal to the coefficients in Eq. 1 for the corresponding equation for nonelectrolytes. The descriptors ***J***^+^ and ***J***^**–**^ and the equation coefficients ***j***^+^ and ***j***^**–**^ refer to cations and anions. For anions ***j***^+^ = 0, for cations ***j***^–^ = 0 and for nonelectrolytes ***j***^+^ = ***j***^**–**^ = 0, and Eq. 4 then reverts to Eq. 1. The ***j***^+^ and ***j***^**–**^ coefficients in Eq. 4 are given in Table 1.

4 $$ { \log }_{ 10}\; P = {\user2{c}} + {\user2{eE}} + {\user2{sS}} + {\user2{aA}} + {\user2{bB}} + {\user2{vV}} + {\user2{j}}^{ + }\; {\user2{J}}^{+}   + {\user2{j}}^{-}\;  {\user2{J}}^{-} $$

Ionic partition coefficients from water to NPOE, or their equivalent as Gibbs energies of transfer, have been determined by a number of workers [19–21]. As we have previously pointed out, experimental values can only be obtained for neutral combinations of ions, e.g. (K^+^ + Cl^–^), and single-ion values have to be referred to some particular convention. Usually the TATB convention [29–31] is used, with log_10_ *P*(Ph_4_P^+^) or log_10_ *P*(Ph_4_As^+^) = log_10_ *P* (Ph_4_B^–^). The various conventions that have been put forward in order to obtain single-ion values have been evaluated [30, 31] and the TATB convention selected as the recommended one. All our studies have used this convention, and this is the convention that Wilke and Zerihun [19] and Samec et al. [21] have used. Gulabowski et al. [20] in their determination of partition coefficients of anions used the decamethylferrocene/decamethylferrocinium couple as a standard, and so for consistency all their ionic partition coefficients had to be converted to the TATB convention. The descriptors for the ions [7] are in Table 3, and the data on ionic partition coefficients [19–21] are in Table 4.

**Table 3:** Descriptors for the ions and ionic species

Ionic species	***E***	***S***	***A***	***B***	***V***	***J*** ^+^	***J*** ^−^
Cs^+^	0.100	2.600	1.170	0.000	0.177	0.438	0.000
Me_4_N^+^	− 0.100	1.310	0.680	0.000	0.764	1.235	0.000
Et_4_N^+^	− 0.100	1.850	0.510	0.000	1.357	1.475	0.000
Pr_4_N^+^	− 0.100	2.020	0.420	0.000	1.921	1.552	0.000
Bu_4_N^+^	− 0.100	2.820	0.610	0.000	2.484	1.418	0.000
Ph_4_P^+^	2.220	3.110	0.040	0.920	2.766	0.480	0.000
Ph_4_As^+^	2.220	3.200	0.070	0.910	2.811	0.581	0.000
Cl^−^	0.100	3.520	0.000	2.320	0.228	0.000	2.363
Br^−^	0.170	2.740	0.000	1.820	0.307	0.000	1.567
I^−^	0.380	3.550	0.000	1.340	0.408	0.000	1.251
ClO_4_^−^	− 0.160	5.140	0.000	0.990	0.493	0.000	1.290
NO_3_^−^	0.170	1.980	0.000	1.970	0.320	0.000	1.703
SCN^−^	0.400	3.380	0.000	1.240	0.365	0.000	1.242
Phenoxide^−^	0.955	2.80	0.00	2.12	0.7536	0.000	1.6760
2-Nitrophenoxide^−^	1.165	2.95	0.00	2.20	0.9278	0.000	1.7200
3-Nitrophenoxide^−^	1.200	3.80	0.00	2.25	0.9278	0.000	2.0600
4-Nitrophenoxide^−^	1.220	4.85	0.00	2.09	0.9278	0.000	2.2000
2,4-Dinitrophenoxide^−^	1.350	6.37	0.00	2.22	1.1020	0.000	2.3907
2,5-Dinitrophenoxide^−^	1.410	4.65	0.00	2.19	1.1020	0.000	1.9631
Picrate^−^	1.580	7.32	0.00	1.67	1.2762	0.000	2.6100
Benzoate^−^	0.880	3.64	0.00	2.88	0.9102	0.000	2.3950
3-Chlorobenzoate^−^	0.990	3.13	0.00	2.57	1.0326	0.000	2.0340
4-Chlorobenzoate^−^	0.990	3.18	0.00	2.58	1.0326	0.000	2.0990
4-Bromobenzoate^−^	1.150	3.43	0.00	2.62	1.0852	0.000	2.2203
4-Iodobenzoate^−^	1.460	3.29	0.00	2.61	1.1684	0.000	2.0590
BPh_4_^−^	1.950	2.720	0.180	1.150	2.700	0.000	− 0.188

**Table 4:** Calculated and observed values of log_10_ *P*_npoe_ for ions and ionic species

	log_10_ *P*_npoe_ (calc.)	log_10_ *P*_npoe_ (obs.)			
		Taken	Ref. 19	Ref. 20	Ref. 21
Cs^+^	− 3.950	− 4.280	− 4.280		− 3.680
Me_4_N^+^	− 2.175	− 2.370	− 2.370		− 1.870
Et_4_N^+^	− 0.482	− 0.440	− 0.440		− 0.460
Pr_4_N^+^	1.470	1.560	1.590		1.520
Bu_4_N^+^	3.004				4.080
Ph_4_P^+^	5.167				5.310
Ph_4_As^+^	5.023	5.340	5.340		5.480
Cl^−^	− 8.688	− 8.690	− 8.690	− 9.220	
Br^−^	− 6.380		− 7.240		
I^−^	− 4.483	− 4.700	− 4.700	− 4.910	
ClO_4_^−^	− 3.824		− 2.960	− 3.100	− 2.700
NO_3_^−^	− 6.530	− 6.290	− 6.290	− 6.580	
SCN^−^	− 4.154	− 4.400	− 4.400	− 4.600	
Phenoxide^−^	− 5.466	− 5.613	− 0.300	− 5.613	
2-Nitrophenoxide^−^	− 5.079	− 5.343		− 5.343	
3-Nitrophenoxide^−^	− 5.485	− 5.473		− 5.473	
4-Nitrophenoxide^−^	− 5.244	− 5.203		− 5.203	
2,4-Dinitrophenoxide^−^	− 5.653	− 5.478		− 5.478	
2,5-Dinitrophenoxide^−^	− 4.917	− 4.783		− 4.783	
Picrate^−^	− 3.031	− 2.803	− 0.300	− 2.803	
Benzoate^−^	− 8.032			− 5.510	
3-Chlorobenzoate^−^	− 6.228	− 6.368		− 6.368	
4-Chlorobenzoate^−^	− 6.261	− 6.118		− 6.118	
4-Bromobenzoate^−^	− 6.190	− 6.488		− 6.488	
4-Iodobenzoate^−^	− 5.667	− 5.548		− 5.548	
BPh_4_^−^	4.748	5.340	5.340		5.310

## Results

All the data that we need to construct Eq. 1 for the water-to-NPOE system are in Table 2. One solute, 4-bromobenzoic acid, was an outlier, and for the remaining 87 solutes we obtained Eq. 5:

5 $$ \begin{aligned}& { \log }_{ 10}\;P_{\text{npoe}} = 0.182+ 0.63 1{\user2{E}} - 0. 4 4 7{\user2{S}}- 2. 2 5 4{\user2{A}} - 3. 9 7 3{\user2{B}} + 3. 5 5 9{\user2{V}} \hfill \\ &N = { 87},\;SD = 0. 2 8 2,\;R^{ 2} = 0. 9 5 5,\;F = { 343}.8,\;{\text{PRESS }} = { 7}. 5 5 2 8,\;Q^{ 2} =  0. 9 4 7,\;\textit{PSD} = 0. 30 4\hfill \\ \end{aligned} $$

The outlier, 4-bromobenzoic acid, had an observed value of log_10_ *P*_npoe_ as 0.82; observed values for 4-chlorobenzoic acid and 4-iodobenzoic acid are 0.88 and 1.46, and so the observed value for 4-bromobenzoic acid does seem to be out of line. In Eq. 5, *N* is the number of solutes, *SD* is the regression standard deviation, *R* is the correlation coefficient, *F* is the *F*-statistic, *PRESS* and *Q*^2^ are the leave-one-out statistics and *PSD* is the predictive standard deviation [32].

Values of the gas to NPOE partition coefficient were obtained through Eq. 3 and are listed in Table 2. Application of the LFER Eq. 2 leads to Eq. 6. As before, the solute 4-bromobenzoic acid was left out.

6 $$ \begin{aligned}& { \log }_{ 10} \;K_{\text{npoe}} = -\,0. 10 4+ 0. 2 90{\user2{E}} + 1. 3 3 3{\user2{S}} + 1. 30 6{\user2{A}} + 0. 9 6 7{\user2{B}} + 0. 7 5 9{\user2{L}} \hfill \\ & N = { 87},\;\textit{SD} = 0. 2 8 2,\;R^{ 2} = 0. 9 9 5,\;F = { 2977}. 2,\;{\text{PRESS }} = { 7}. 4 6 3 9,\;Q^{ 2} =  0. 9 9 4,\;\textit{PSD} = 0. 30 3\hfill \\ \end{aligned} $$

Details of observed values of log_10_ *K*_npoe_ for ions and ionic species are in Table 4. There are a number of discrepancies between the sets of data [19, 21], and so we took the values of Wilke and Zerihun [19] for consistency, and supplemented these with data on anions from Gulabowski et al. [20]. In Eq. 4 the coefficients ***c, e***, ***s***, ***a***, ***b*** and ***v*** are taken as the same as those for the equation for neutral species, Eq. 5, and so there are only two coefficients to be determined. The full equation is shown as Eq. 7:

7 $$ { \log }_{ 10}\;P_{\text{npoe}} = 0.182 + 0.631{\user2{E}} - 0. 4 4 7{\user2{S}} - 2. 2 5 4{\user2{A}} - 3. 9 7 3 {\user2{B}} + 3. 5 5 9 {\user2{V}} - 2. 3 4 2{\user2{J}}^{ + } + 0. 4 4 4{\user2{J}}^{-} $$

**Fig. 1:**
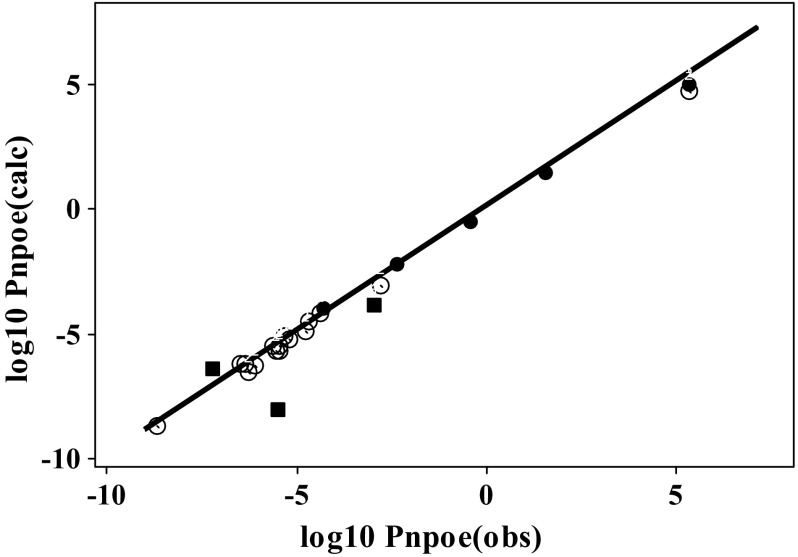
Plot of log_10_ *P*_npoe_(calc.) against log_10_ *P*_npoe_(obs.): ○ anions, ● cations, the three outliers ■. The regression line for neutral solutes is shown as __________

The usual statistics, as in Eqs. 5 and 6, do not apply to Eq. 7 because the coefficients ***c, e***, ***s***, ***a***, ***b*** and ***v*** are fixed. However, for the 21 ions, Eq. 7 fits values of log_10_ *P*_npoe_ with an SD of only 0.236 log_10_ units. The observed values that we used and the calculated values are in Table 4. We left out data on the bromide anion, the perchlorate anion and the benzoate anion, that were very considerably out of line. It is perhaps not surprising that we find a number of values of log_10_ *P*_npoe_ for ions and ionic species to be out of line, considering the differences in some of the experimental values: 0.60 for Cs^+^ and 0.50 for Me_4_N^+^ (Table 4). The calculated values of log_10_ *P*_npoe_ for the ions show no systematic deviations, as can be seen from Fig. 1.

## Discussion

The equations for log_10_ *P*_npoe_ and log_10_ *K*_npoe_ for 87 neutral species are consistent with previous such equations for partition from water and the gas phase to solvents. The *SD* values in both Eqs. 5 and 6 are rather larger than those we usually find, but the 87 compounds include a large number of drugs for which it is more difficult to obtain descriptors. The values of the coefficients in Eqs. 5 and 6 are not particularly unusual, although the ***b***-coefficient in Eq. 6 (0.967) indicates that NPOE has some hydrogen bond acidity. This can hardly be due to the presence of water in water-saturated NPOE because the solubility of water in NPOE is only 0.046 mol·dm^−3^ [21], less than the water solubility in nitrobenzene and in 1,2-dichloroethane.

Liu et al. [18] suggested that NPOE could replace 1,2-dichloroethane as regards solvation and partition of nonelectrolytes. A simple way of analyzing the coefficients in Eq. 1 is to regard the coefficients ***e***, ***s***, ***a***, ***b*** and ***v*** as points in five-dimensional space. Then the distance, D’, between a point for a particular system and the point for the NPOE system will indicate how near the particular system is to the NPOE system in terms of solubility related properties [33]. Results in terms of the distance parameter D’ [33] are in Table 5, in order of the value of D’. We give also in Table 5, details of the nature of the various water–solvent systems. For many of the water–aprotic solvent systems solubilities in the dry solvent are effectively the same as those in the solvent equilibrated with water; we denote these as w/d. The solvents for which data were obtained only using the dry solvent are denoted as ‘d’. Wet octanol is an exception in that the solubility properties of water-saturated octanol, ‘w’, are not the same as those of dry octanol. The solvent systems with nitrobenzene or 1,2-dichloroethane are chemically the closest to the NPOE system. Abraham and Martins [33] suggested that for two systems to be regarded as similar the distance parameter should not be larger than about 0.5–0.8 units. The D’ values for nitrobenzene and 1,2-dichloroethane are slightly outside this criterion, at 0.981 and 0.999 respectively, but the water–NPOE system is still closer to the nitrobenzene or 1,2-dichloroethane systems than to any of the other systems in Table 5.

**Table 5:** The five-dimensional distance parameter, D’, of systems from the water–NPOE system, Eq. 1, and the gas–NPOE system, Eq. 2

Solvent system	System	D’ (Eq. 1)	D’ (Eq. 2)
NPOE (this work)	w/d	0.000	0.000
Nitrobenzene	w/d	0.981	0.709
1,2-Dichloroethane	w/d	0.999	0.939
Benzonitrile	w/d	1.168	1.269
Acetonitrile	d	1.261	1.722
Propylene carbonate	d	1.506	1.773
Chlorobenzene	w/d	1.604	1.484
Nitromethane	w/d	1.687	1.782
Propanone	d	1.916	2.154
Sulfolane	d	2.340	2.204
Tetrahydrofuran	d	2.419	2.309
Wet octan-1-ol	w	2.436	2.325
Butan-1-ol	d	2.438	2.536
Hexan-1-ol	d	2.487	2.489
Methanol	d	2.612	2.632
Propan-1-ol	d	2.621	2.706
Ethanol	d	2.694	2.692
t-Butanol	d	2.731	2.893
Propan-2-ol	d	2.787	2.870
Dimethylformamide	d	3.095	3.005
1,2-Propylene glycol	d	3.117	3.093
*N*-Methylpyrrolidinone	d	3.285	3.402
Formamide	d	3.303	3.151
Ethylene glycol	d	3.368	3.467
Dmethylacetamide	d	3.582	3.408
Dimethylsulfoxide	d	3.786	4.193

We can also calculate the D’ parameter using the coefficients ***e***, ***s***, ***a***, ***b*** and ***l*** from Eq. 2. These are also given in Table 5. Note that the entries are in order of the solvents—this is not quite the same as the order of D’ from Eq. 1, although the order in terms of Eq. 2 follows very closely the order in terms of Eq. 1. Again, NPOE is a better model for nitrobenzene and 1,2-dichloroethane than any of the other solvents listed, as regards nonelectrolytes.

In order to analyze partition of ionic species, we could calculate D’ using the coefficients in Eq. 4. However, the results would be dominated by the ‘nonelectrolyte’ coefficients ***e***, ***s***, ***a***, and ***b*** and would yield little direct information on partition of ionic species. A direct method of assessing the various water–solvent systems in terms of ionic species is simply to survey the actual ionic partition coefficients from water to the various solvents. Unfortunately, there are very few ionic species for which partition coefficients are known across any reasonable number of systems. We can circumvent this difficulty by using the data in Tables 1 and 3 to calculate values of ionic partition coefficients for a number of representative systems as shown in Table 6. We also give the *SD* for the values of log_10_ *P* against the NPOE system as the standard. From the *SD* values, it can be seen that NPOE would be a good substitute for 1,2-dichloroethane and possibly for nitrobenzene as regards partition of ionic species. This is a quite important result. Seip et al. [34] have set out a classification of solvents in terms of their suitability as supported liquid membranes in electromembrane extraction. NPOE was rated in the highest category, whereas nitrobenzene was classed as unsuitable.

**Table 6:** Calculated values of log_10_ *P* for ions and ionic species from water to various solvents

	NPOE	12DCE	PhCN	PhNO_2_	Propanone	PhCl	BuOH	Wet Oct^a^
Cs^+^	− 3.950	− 4.175	− 2.081	− 3.354	− 0.986	− 6.146	− 3.221	− 3.205
Et_4_N^+^	− 0.482	− 0.908	0.800	− 0.537	1.723	− 2.989	− 1.546	− 1.184
Pr_4_N^+^	1.470	1.413	3.015	1.787	3.802	− 0.540	0.279	0.551
Bu_4_N^+^	3.004	3.589	5.393	4.220	6.118	1.647	2.243	2.269
Cl^−^	− 8.688	− 9.320	− 9.011	− 7.450	− 10.026	− 15.094	− 5.269	− 4.627
I^−^	− 4.483	− 4.257	− 3.885	− 3.007	− 4.661	− 7.914	− 3.567	− 3.293
ClO_4_^−^	− 3.824	− 2.772	− 2.000	− 1.202	− 3.020	− 6.969	− 3.557	− 3.636
NO_3_^−^	− 6.530	− 7.191	− 7.204	− 6.092	− 7.843	− 11.215	− 3.699	− 3.105
Phenoxide	− 5.466	− 5.900	− 5.874	− 4.494	− 6.703	− 10.007	− 3.126	− 2.463
4-Nitro	− 5.244	− 5.253	− 4.767	− 2.917	− 5.998	− 10.808	− 2.862	− 2.355
Benzoate	− 8.032	− 8.659	− 8.584	− 6.666	− 9.767	− 14.391	− 4.450	− 3.568
4-Bromo	− 6.190	− 6.700	− 6.652	− 4.776	− 7.745	− 11.890	− 2.861	− 2.079
4-Nitro	− 6.909	− 7.445	− 7.431	− 5.549	− 8.568	− 12.721	− 3.635	− 2.771
*SD*		0.555	1.247	1.437	1.899	4.654	2.416	2.936

^a^Wet octan-1-ol

Davis and Di Toro [16] have approached the problem of descriptors for ionic species rather differently from the methods we have employed. They use quantum calculated partitions into a large number of solvents and define ionic species in terms of five descriptors only. However, they then require different equations for partition of neutral molecules, anions and cations from water into a given solvent. Davis and Di Toro [16] set out equations for the partition of anions into propanone, acetonitrile, methanol and dimethylsulfoxide with an *RMSE* in log_10_ *P* from 0.39 to 0.51, and an equation for partition of cations from water to octanol with an *SD* of 1.16 in log_10_ *P*. At the moment the two methods are independent of each other, although it would be useful if descriptors for ionic species could somehow be interchanged.

## Conclusions

We have constructed LFERs for partition from water to NPOE and from the gas phase to NPOE for 87 neutral solutes. The latter equation is new and has not been set out before. The equations reveal that the solution properties of NPOE for nonelectrolyte solutes are quite similar to solution properties of the typical aprotic solvents 1,2-dichloroethane and nitrobenzene. Almost the same result is obtained by the examination of partition coefficients for ions and ionic species. The solution properties of NPOE for electrolytes are quite close to those of 1,2-dichloroethane although a little way away from aprotic solvents such as nitrobenzene and benzonitrile.
